# Optimal Sensor Placement in Buildings: Stationary Excitation

**DOI:** 10.3390/s25247470

**Published:** 2025-12-08

**Authors:** Farid Ghahari, Daniel Swensen, Hamid Haddadi

**Affiliations:** 1 California Geological Survey, Sacramento, CA 95814, USA; 2 The B. John Garrick Institute for the Risk Sciences, University of California, Los Angeles, CA 90095, USA

**Keywords:** optimal sensor placement, buildings, gaussian process regression, stationary response

## Abstract

This study presents a methodology for determining the optimal placement of sensors along the height of buildings to minimize uncertainty in reconstructing structural response at non-instrumented floors. Recent advancements in sensing technology have expanded the application of sensor data in earthquake and structural engineering, including model validation, post-event damage assessment, and structural health monitoring. However, to lower the costs of sensor installation and maintenance—particularly at the regional scale—it is essential to strategically place sensors to maximize the value of the collected data. Because the optimal sensor configuration depends on the specific objectives of an instrumentation project, there is no universal solution to the sensor placement problem. In this study, we focus on identifying sensor locations that allow for accurate interpolation of structural responses at non-instrumented floors with minimal prediction uncertainty. This objective supports the primary goal of the California Strong Motion Instrumentation Program (CSMIP), which is to collect structural response data with the highest possible accuracy and the lowest uncertainty. The proposed method is limited to stationary excitations (e.g., ambient vibrations or distant earthquakes) and to buildings with uniform mass and stiffness distributions along their height. Under these assumptions, a Gaussian Process Regression (GPR) model is used to quantify response prediction uncertainty and minimize the total uncertainty across the building height by placing sensors at the most informative locations. The GPR model is based on a simple shear-flexural beam representation, which effectively approximates the building using very few parameters—parameters that can be estimated from limited building information. The proposed method is verified and validated using both simulated and real data. Finally, a table is proposed that can be used by strong motion networks to facilitate more quantitative decision-making regarding sensor placement. While assumptions used in this study may seem restrictive, they strike a practical balance between accuracy and simplicity for large-scale applications such as CSMIP. The extension of this work to non-stationary excitations and general building types by training the GPR model on recorded seismic data rather than random vibration theory is under development.

## 1. Introduction

**Figure 1:**
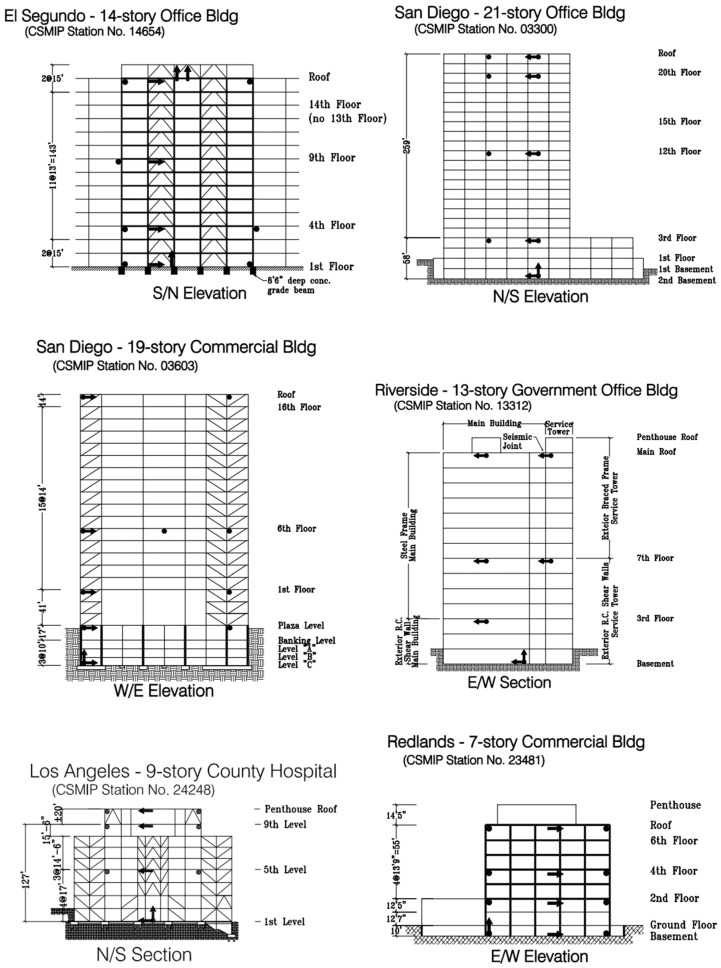
Examples of CSMIP buildings with sparse instrumentation. Arrows and circles represent the sensors.

The California Strong Motion Instrumentation Program (CSMIP) in the California Geological Survey (CGS) was established in 1972, after the devastating 1971 San Fernando earthquake to obtain vital earthquake data and improve understanding of the engineering and scientific communities about earthquakes and also how civil structures respond to ground shaking [1]. Currently, the program has more than 1390 active stations throughout California including 946 ground-response stations, 273 buildings, 26 dams, and 82 bridges with the total number of sensors exceeding 10,200. While post-earthquake damage assessment was one of its original objectives, Structural Health Monitoring (SHM) was in its infancy during CSMIP’s initiation. Consequently, buildings, especially mid- to high-rise structures, were—and, to a large extent, still are—equipped with a limited number of sensors due to the sensor installation and maintenance cost (see Figure 1). Although more extensive instrumentation is recommended in some guidelines, such as the Los Angeles Tall Buildings Guidelines [2], it is not yet feasible to install sensors on every floor of a building due to instrumentation and maintenance costs.

Recent advancements over the past two decades in both software and hardware have made vibration-based post-event (e.g., earthquake) damage assessment and continuous structural health monitoring (SHM) not only feasible but essential for advancing a resilient built environment [3,4,5,6]. These capabilities were previously unattainable through traditional, labor-intensive visual in situ inspections. Among the most common approaches to post-event assessment are data-driven modal identification methods [7,8,9] and model-based system identification techniques [10,11,12,13,14,15], which utilize data from instrumented structures to evaluate structural health and performance either after an earthquake or during regular operation. However, as previously noted, most instrumented structures are sparsely instrumented due to the high costs associated with installation and maintenance. This limited instrumentation introduces substantial uncertainty into response estimation [16,17,18].

Deterministic interpolation techniques, such as cubic splines [19], have been used to estimate structural responses at non-instrumented floors [20,21,22]. To address the inability of these methods to quantify uncertainty systematically, we recently introduced a hybrid deterministic-probabilistic approach [23]. This method combines a simple beam model—easily calibrated by adjusting a few parameters [24,25,26,27]—with a Gaussian Process Regression (GPR) model [28,29], a non-parametric probabilistic technique widely used across various fields, including earthquake engineering (e.g., [30]). Through extensive verification and real-world case studies, this approach has been shown to yield highly accurate mean estimations while simultaneously quantifying associated uncertainties—an essential feature when synthetic response data are intended for probabilistic post-event damage assessment, such as FEMA P-58 [31] methodologies [32].

Following the development of this hybrid approach [23], a new project was initiated within the CSMIP, aiming to minimize estimation and interpolation uncertainty by optimizing sensor placement along the height of building structures. While this approach may not enhance existing instrumented structures, CGS has recently launched an extensive initiative to either fully re-instrument or add additional sensors to more than 70 buildings—making the findings of this sensor placement study especially relevant. Furthermore, the number of CSMIP-instrumented buildings continues to grow, as California’s seismic safety code requires seismic monitoring systems in tall buildings [2].

Optimal sensor placement has long been a subject of research in fields where data collection is essential [33,34,35,36,37,38]. In earthquake and structural engineering, interest in this topic dates back to the early years of structural vibration monitoring (see, e.g., [39,40]) and was revitalized around the year 2000 by pioneering work led by Beck and his former students [41,42,43,44,45,46]. With the exception of Limongelli’s study [47], which used a deterministic approach to minimize the difference between the response reconstructed via cubic spline interpolation and the true response for a series of typical building models, information theory forms the core of all these optimal sensor placement strategies, with differences among studies arising from the specific information metric used. Examples include the Fisher Information Matrix [39], information entropy [41], joint entropy [48], value of information [49,50], Kullback–Leibler divergence [51,52], and mutual information [45].

In addition to the information metric, the objective of the sensor placement significantly influences the results. Common objectives in the literature include finite element model updating, parameter estimation, model selection, modal identification, damage detection, and structural health monitoring. Although response reconstruction has been considered among these objectives (e.g., [46]), it is often treated as secondary to model updating. However, CSMIP’s primary mandate is to provide structural response data following earthquakes. As such, response measurement and reconstruction are central objectives.

Building on the work of Papadimitriou et al. [43] and Gorodesky and Marzouk [53], we propose an optimal sensor placement strategy for building structures that minimizes uncertainty in response prediction at non-instrumented floors using Gaussian Process Regression (GPR). Although many studies have used finite element models combined with optimization techniques for this purpose (see, e.g., [54,55]), the GPR approach offers greater flexibility for large-scale problems such as CSMIP buildings, for which developing detailed numerical models may not be feasible. The methodology is based on two key assumptions: (1) the excitation is stationary, and (2) the building is accurately modeled as a shear-flexural coupled beam. These assumptions allow us to apply random vibration theory in conjunction with beam theory to analytically construct the covariance kernel function used in the GPR model. While these assumptions may seem limiting, as structures can exhibit nonlinear and non-stationary behavior [56], they provide a balance between accuracy and parsimony that is necessary for large-scale applications such as CSMIP. Moreover, an extension of this method to non-stationary excitations is under development, in which GPR will be trained using recorded seismic data instead of random vibration theory. Although any technique has its own advantages and drawbacks, this future extension will specifically address two limitations of the current study: the assumption of stationary excitation and the restriction to beam-type buildings.

This study differs from the work of Papadimitriou et al. [43] in two ways: (1) a shear-flexural coupled beam is used here instead of a purely flexural model; (2) the formulation is derived for boundary conditions compatible with building structures. Moreover, the proposed methodology is validated using real earthquake data, whereas the work of Papadimitriou et al. [43] is limited to numerical examples of non-seismic cases.

In the next section, we first provide a brief review of GPR. Then, using the beam model, we derive the analytical kernel function for a clamped shear-flexural beam, representative of tall building structures. Finally, we introduce the objective function used to determine the optimal sensor locations along the height of the building. A series of case studies is conducted to identify and verify optimal sensor placements for buildings with varying numbers of sensors. These results are compiled into a practical reference table. We also validate our proposed sensor placement strategy using earthquake data from an actual instrumented building. The major findings of this study are summarized in the Section 5.

It should be emphasized that the scope of this study is limited to optimizing the locations of a specified number of sensors along the height of building structures. Determining the number of sensors required to meet specific criteria is beyond the scope of this study, although it can be inferred from the results, as will be discussed later. Furthermore, all sensors are assumed to be flawless in this study, while the effects of sensor faults and measurement quality have been investigated in other studies (e.g., [57]).

## 2. The Proposed Approach

### 2.1. Gaussian Process Regression

Gaussian Process Regression (GPR) is a Bayesian approach for function approximation. Instead of providing a single functional value $f\left(x\right)$, GPR yields posterior probability distribution over all possible functions [29]. By definition, a GP consists of a set of random variables, any finite subset of which follows a joint Gaussian distribution. Consequently, its prior can be characterized by a prior mean function $m_{0}\left(x\right)$ and a covariance kernel $k\left(x,\prime x^{\prime };\vartheta \right)$, as follows

(1) $$f\left(x\right)\sim GP\left(m_{0}\left(x\right),k\left(x,x^{\prime };\vartheta \right)\right),$$

with

(2) $$m_{0}\left(x\right)=E\left[f\left(x\right)\right],$$

(3) $$k\left(x,x^{\prime };\vartheta \right)=E\left[\left(f\left(x\right)-m_{0}\left(x\right)\right)\left(f\left(x^{\prime }\right)-m_{0}\left(x^{\prime }\right)\right)\right],$$

where $E\left[.\right]$ denotes the expected value, and ϑ is a vector of hyperparameters that define the covariance kernel. In this study, we assume that both the parameter x and function value $f\left(x\right)$ are scalar. Additionally, while not necessary, the prior mean is often assumed to be zero, as any known nonzero trend can be subtracted from the data.

Now, assume that noisy function values are available at $n_{d}$ parameter values, given by $\bar{x}={\left[\begin{array}{cccc} {\bar{x}}_{1} & {\bar{x}}_{2} & \ldots & {\bar{x}}_{n_{d}} \end{array}\right]}^{T}$ where the superscript T denotes the matrix (or vector) transpose. That is, $\bar{y}=f\left(\bar{x}\right)+\epsilon$ where $f\left(\bar{x}\right)={\left[\begin{array}{cccc} f\left({\bar{x}}_{1}\right) & f\left({\bar{x}}_{2}\right) & \ldots & f\left({\bar{x}}_{n_{d}}\right) \end{array}\right]}^{T}$ is the vector of true function values, $\bar{y}={\left[\begin{array}{cccc} {\bar{y}}_{1} & {\bar{y}}_{2} & \ldots & {\bar{y}}_{n_{d}} \end{array}\right]}^{T}$ is the vector of noisy measurement, and $\epsilon ={\left[\begin{array}{cccc} \epsilon_{1} & \epsilon_{2} & \ldots & \epsilon_{n_{d}} \end{array}\right]}^{T}$ is the vector of measurement noise. Based on the aforementioned definition of a GP, function value at any arbitrary input value of $\hat{x}$ along with the measurement data construct a joint Gaussian distribution. Thus,

(4) $$\left[\begin{array}{c} \bar{y}\\ f\left(\hat{x}\right) \end{array}\right]\sim N\left(\left[\begin{array}{c} 0\\ 0 \end{array}\right],\begin{array}{cc} C\left(\bar{x},{\bar{x}}^{\prime };\vartheta \right)+\sigma_{0}^{2}I & k\left(\bar{x},\hat{x};\vartheta \right)\\ {k\left(\bar{x},\hat{x};\vartheta \right)}^{T} & k\left(\hat{x},\hat{x};\vartheta \right) \end{array}\right),$$

where the matrix $C\left(\bar{x},{\bar{x}}^{\prime };\vartheta \right)$ is the covariance matrix which is calculated using the covariance kernel as

(5) $$C\left(\bar{x},{\bar{x}}^{\prime };\vartheta \right)=\left[\begin{array}{cccc} k\left({\bar{x}}_{1},{\bar{x}}_{1};\vartheta \right) & k\left({\bar{x}}_{1},{\bar{x}}_{2};\vartheta \right) & \ldots & k\left({\bar{x}}_{1},{\bar{x}}_{n_{d}};\vartheta \right)\\ k\left({\bar{x}}_{2},{\bar{x}}_{1};\vartheta \right) & k\left({\bar{x}}_{2},{\bar{x}}_{2};\vartheta \right) & \vdots & k\left({\bar{x}}_{2},{\bar{x}}_{n_{d}};\vartheta \right)\\ \vdots & \ldots & \ddots & \vdots \\ k\left({\bar{x}}_{n_{d}},{\bar{x}}_{1};\vartheta \right) & k\left({\bar{x}}_{n_{d}},{\bar{x}}_{2};\vartheta \right) & \ldots & k\left({\bar{x}}_{n_{d}},{\bar{x}}_{n_{d}};\vartheta \right) \end{array}\right],$$

and $k\left(\bar{x},\hat{x};\vartheta \right)$ is a vector representing the correlation between $f\left(\bar{x}\right)$ and $f\left(\hat{x}\right)$, that is

(6) $$k\left(\bar{x},\hat{x};\vartheta \right)=\left[\begin{array}{c} k\left(x_{1},\hat{x};\vartheta \right)\\ k\left(x_{2},\hat{x};\vartheta \right)\\ \vdots \\ k\left(x_{n_{d}},\hat{x};\vartheta \right) \end{array}\right].$$

The $\sigma_{0}^{2}I_{n_{d}\times n_{d}}$ term added to the covariance matrix of the measured data is representative of the measurement noise which is assumed to be a zero-mean uncorrelated white noise with a variance of $\sigma_{0}^{2}$. Note that $I_{n_{d}\;\times \;n_{d}}$ denotes the identity matrix of size $n_{d}$, which is a square matrix with ones on the main diagonal and zeros elsewhere.

**Figure 2:**
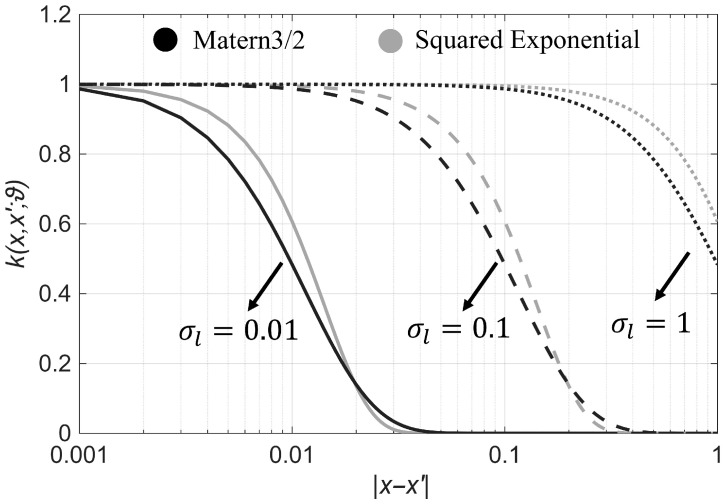
The Squared Exponential and Matern 3/2 kernel functions with three different length-scale values of 0.01, 0.1, and 1, and a constant signal variance of 1.

It is then mathematically straightforward to show that the conditional distribution of $f\left(\hat{x}\right)$ given measurements ($\bar{x}$, $\bar{y}$) is

(7) $$\left.f\left(\hat{x}\right)\right|\bar{x},\bar{y}\sim N\left(m_{f\left(\hat{x}\right)},\sigma_{f\left(\hat{x}\right)}^{2}\right),$$

where

(8) $$m_{f\left(\hat{x}\right)}={k\left(\bar{x},\hat{x};\vartheta \right)}^{T}{\left[C\left(\bar{x},{\bar{x}}^{\prime };\vartheta \right)+\sigma_{0}^{2}I\right]}^{-1}\bar{y},$$

(9) $$\sigma_{f\left(\hat{x}\right)}^{2}=k\left(\hat{x},\hat{x};\vartheta \right)-{k\left(\bar{x},\hat{x};\vartheta \right)}^{T}{\left[C\left(\bar{x},{\bar{x}}^{\prime };\vartheta \right)+\sigma_{0}^{2}I\right]}^{-1}k\left(\bar{x},\hat{x};\vartheta \right),$$

are posterior mean and variance of the prediction at $\hat{x}$. To apply the equations above, in addition to the data, the covariance kernel and its hyperparameters are required. The common practice is to select an appropriate covariance kernel from a wide range of available functions and then find the optimal hyperparameter values by minimizing the negative log-likelihood of the data as shown below [58]

(10) $$NLL\left(\bar{y}\right)=\frac{1}{2}{\bar{y}}^{T}{\left[C\left(\bar{x},{\bar{x}}^{\prime };\vartheta \right)+\sigma_{0}^{2}I\right]}^{-1}\bar{y}+\frac{1}{2}\log\left|\left(C\left(\bar{x},{\bar{x}}^{\prime };\vartheta \right)+\sigma_{0}^{2}I\right)\right|+\frac{n_{d}}{2}\log2\pi ,$$

in which $\left|.\right|$ denotes the matrix determinant. As an example, Figure 2 compares the Squared Exponential (SE) and Matern 3/2 kernels [59], which are defined by the following equations,

(11) $$k_{SE}\left(x,x^{\prime };\vartheta \right)=\sigma_{f}^{2}\;e^{-\frac{d^{2}}{2\sigma_{l}^{2}}},$$

(12) $$k_{M\frac{3}{2}}\left(x,x^{\prime };\vartheta \right)=\sigma_{f}^{2}\left(1+\frac{\sqrt{3}d}{\sigma_{l}}\right)e^{-\frac{\sqrt{3}d}{\sigma_{l}}}.$$

where $d=\left|x-x^{\prime }\right|$ and $\vartheta ={\left[\sigma_{f}^{2},\sigma_{l}\right]}^{T}$, in which $\sigma_{f}^{2}$ and $\sigma_{l}$ are two hyperparameters called the signal variance and correlation length, respectively. As seen in Figure 2, assuming a signal variance of 1, a small correlation length leads to a rapid drop in correlation, indicating minimal similarity for distances greater than this length. Conversely, a large correlation length implies nearly identical function values across the domain.

For the optimal sensor placement task, data is not usually available a priori to use with Equation (10). Therefore, we represent the structure with a simple model and use an analytical solution to determine the covariance kernel, making certain assumptions about the statistical properties of the external loads. This approach was previously used by Papadimitriou et al. [43] with a flexural beam under simply supported boundary conditions, which are not applicable to building structures. In this study, we reproduce the kernel function using the cantilever coupled beam model [24,25,26,60] under spatially deterministic (uniform) but temporally uncorrelated lateral loads.

### 2.2. Analytical Covariance Kernel Using Beam Model

**Figure 3:**
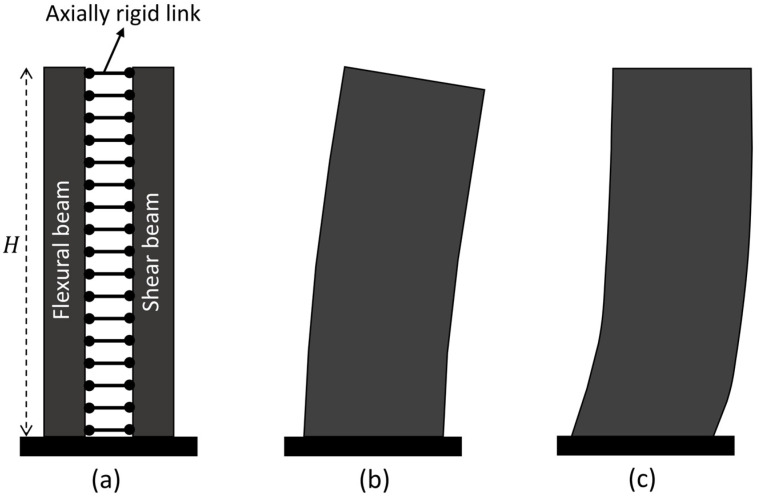
(**a**) Flexural-shear beam model to represent multi-story building structures. Pure flexural and shear behaviors are shown in (**b**,**c**) (this figure is adapted from [60]).

Figure 3a shows the beam model used in this study, which combines flexural and shear beams, shown in Figure 3b and 3c, respectively. Assuming a uniform mass per unit length ρ, shear stiffness GA, and flexural stiffness EI along the beam height L, the governing partial differential equation of this model under lateral force $p\left(x,t\right)$ is given as [26]

(13) $$\rho \frac{\partial^{2}u\left(x,t\right)}{\partial t^{2}}+\frac{EI}{L^{4}}\frac{\partial^{4}u\left(x,t\right)}{\partial x^{4}}-\frac{GA}{L^{2}}\frac{\partial u\left(x,t\right)}{\partial x}=p\left(x,t\right),$$

where $u\left(x,t\right)$ represents lateral displacement response of the beam at normalized height x=h/L (0≤x≤1) with h being the physical height measured from the base. Dividing both sides of the equation above by EI, we can write

(14) $$\frac{\rho }{EI}\frac{\partial^{2}u\left(x,t\right)}{\partial t^{2}}+\frac{1}{L^{4}}\frac{\partial^{4}u\left(x,t\right)}{\partial x^{4}}-\frac{\alpha^{2}}{L^{4}}\frac{\partial u\left(x,t\right)}{\partial x}=\frac{1}{EI}p\left(x,t\right),$$

where $\alpha =L\sqrt{\frac{GA}{EI}}$ is a dimensionless parameter that indicates the degree of relative contributions of flexural and shear behaviors. Using the method of separation of variables, where $u\left(x,t\right)=\sum_{k=1}^{\infty }\varphi_{k}\left(x\right)q_{k}\left(t\right)$, the equation of motion under free vibration conditions can be written as

(15) $$\frac{EI}{\rho L^{4}}\left(\frac{\varphi_{k}^{\prime \prime \prime \prime }\left(x\right)}{\varphi_{k}\left(x\right)}-\alpha^{2}\frac{\varphi_{k}^{\prime \prime }\left(x\right)}{\varphi_{k}\left(x\right)}\right)=-\frac{{\ddot{q}}_{k}\left(t\right)}{q_{k}\left(t\right)}=\omega_{k}^{2},$$

resulting in two sets of separate equations of

(16) $${\ddot{q}}_{k}\left(t\right)+\omega_{k}^{2}q_{k}\left(t\right)=0,$$

(17) $$\varphi_{k}^{\prime \prime \prime \prime }\left(x\right)-\alpha^{2}\varphi_{k}^{\prime \prime }\left(x\right)-\omega_{k}^{2}\frac{\rho L^{4}}{EI}\varphi_{k}\left(x\right)=0.$$

In the equations above, the overdot and the prime (′) denote derivatives with respect to time and space, respectively. Solving the characteristic equation of Equation (17) results in the following real ($\beta_{k}$) and imaginary ($i\gamma_{k}$) roots as

(18) $$\beta_{k}=\sqrt{\frac{1}{2}\alpha^{2}+\frac{1}{2}\sqrt{\alpha^{4}+4\omega_{k}^{2}\frac{\rho L^{4}}{EI}}},$$

(19) $$\gamma_{k}=\sqrt{-\frac{1}{2}\alpha^{2}+\frac{1}{2}\sqrt{\alpha^{4}+4\omega_{k}^{2}\frac{\rho L^{4}}{EI}}},$$

from which it is easy to show that $\beta_{k}^{2}=\alpha^{2}+\gamma_{k}^{2}$. Therefore, the natural frequencies of the beam can be expressed versus $\gamma_{k}$ and $\beta_{k}$ as follows

(20) $$\omega_{k}^{2}=\frac{EI}{\rho L^{4}}\gamma_{k}^{2}{\beta_{k}}^{2}.$$

Having the roots of the characteristic equation, the solution of Equation (17), i.e., mode shapes, can be written as

(21) $$\varphi_{k}\left(x\right)=c_{1}\sin\left(\gamma_{k}x\right)+c_{2}\cos\left(\gamma_{k}x\right)+c_{3}\sinh\left(\beta_{k}x\right)+c_{4}\cosh\left(\beta_{k}x\right).$$

Using four boundary conditions, and solving the system of homogenous equations, $\varphi_{k}\left(x\right)$ can be calculated up to an arbitrary factor $d_{k}$ as

(22) $$\varphi_{k}\left(x\right)=d_{k}\left[\sin\left(\gamma_{k}x\right)-\frac{\gamma_{k}}{\beta_{k}}\sinh\left(\beta_{k}x\right)+\eta_{k}\left[\cosh\left(\beta_{k}x\right)-\cos\left(\gamma_{k}x\right)\right]\right]$$

with

(23) $$\eta_{k}=\frac{\gamma_{k}^{2}\sin\gamma_{k}+\gamma_{k}\beta_{k}\sinh\beta_{k}}{\gamma_{k}^{2}\cos\gamma_{k}+\beta_{k}^{2}\cosh\beta_{k}},$$

and an additional equation

(24) $$2+\left(2+\frac{\alpha^{4}}{\gamma_{k}^{2}{\beta_{k}}^{2}}\right)\cos\left(\gamma_{k}\right)\cosh\left(\beta_{k}\right)+\frac{\alpha^{2}}{\gamma_{k}\beta_{k}}\sin\left(\gamma_{k}\right)\sinh\left(\beta_{k}\right)=0,$$

from which $\gamma_{k}$ that are needed to calculate the natural frequencies in Equation (20), can be determined. To complete the solution, temporal component of the response also needs to be calculated. Instead of Equation (16), which applies to free vibration conditions, we solve Equation (14), having estimated the spatial part of the response. That is,

(25) $$\frac{\rho }{EI}\sum_{k=1}^{\infty }\varphi_{k}\left(x\right){\ddot{q}}_{k}\left(t\right)+\frac{1}{L^{4}}\sum_{k=1}^{\infty }\left(\varphi_{k}^{\prime \prime \prime \prime }\left(x\right)-\alpha^{2}\varphi_{k}^{\prime \prime }\left(x\right)\right)q_{k}\left(t\right)=\frac{1}{EI}p\left(x,t\right).$$

By multiplying both sides of the equation above by $\varphi_{j}\left(x\right)$ and integrating over the length of the beam, we have

(26) $$\rho \sum_{k=1}^{\infty }{\ddot{q}}_{k}\left(t\right)\int_{0}^{1}\varphi_{k}\left(x\right)\varphi_{j}\left(x\right)dx+\frac{EI}{L^{4}}\sum_{k=1}^{\infty }q_{k}\left(t\right)\int_{0}^{1}\varphi_{j}\left(x\right)\left(\varphi_{k}^{\prime \prime \prime \prime }\left(x\right)-\alpha^{2}\varphi_{k}^{\prime \prime }\left(x\right)\right)dx=\int_{0}^{1}\varphi_{j}\left(x\right)p\left(x,t\right)dx,$$

which can be simplified as

(27) $$M_{j}{\ddot{q}}_{j}\left(t\right)+K_{j}q_{j}\left(t\right)=P_{j}\left(t\right),\;\mathrm{for}\;j=1\ldots \infty$$

where

(28) $$M_{j}=\rho \int_{0}^{1}\varphi_{j}^{2}\left(x\right)dx,$$

(29) $$K_{j}=\frac{\alpha^{2}EI}{L^{4}}\int_{0}^{1}{\left[\varphi_{j}^{\prime }\left(x\right)\right]}^{2}dx,$$

(30) $$P_{j}\left(t\right)=\int_{0}^{1}\varphi_{j}\left(x\right)p\left(x,t\right)dx,$$

in which we used the fact that

(31) $$\int_{0}^{1}\varphi_{j}\left(x\right)\varphi_{k}\left(x\right)dx=\left\{\begin{array}{ccc} 0 & \mathrm{for} & j\neq k\\ \int_{0}^{1}\varphi_{j}^{2}\left(x\right)dx & \mathrm{for} & j=k \end{array}\right.\;,$$

(32) $$\int_{0}^{1}\varphi_{j}\left(x\right)\left(\varphi_{k}^{\prime \prime \prime \prime }\left(x\right)-\alpha^{2}\varphi_{k}^{\prime \prime }\left(x\right)\right)=\left\{\begin{array}{ccc} 0 & \mathrm{for} & j\neq k\\ \int_{0}^{1}{\left[\varphi_{j}^{\prime }\left(x\right)\right]}^{2}dx & \mathrm{for} & j=k \end{array}\right.\;.$$

For implementation convenience, the analytical expression of these two integrals is provided below

(33) 1dj2∫01φj2xdx=142−2γj2βj2−2ηjγj+2γjηjβj2+4ηj2+2ηjcos2γjγj−2γjηjcosh2βjβj2−8γj1+ηj2coshβjsinγjβj2+γj2+ηj2−1sin2γjγj+8γj2−βj2ηj2cosγj sinhβjβjβj2+γj2+8ηjsinγjsinhβjβj+γj2+βj2ηj2sinh2βjβ3

(34) $$\begin{array}{c} \frac{1}{d_{j}^{2}}\int_{0}^{1}{\left[\varphi_{j}^{\prime }\left(x\right)\right]}^{2}dx=\frac{1}{4}(-4\gamma_{j}\eta_{j}-2\beta_{j}^{2}\eta_{j}^{2}+2\gamma_{j}^{2}\left(2+\eta_{j}^{2}\right)-2\gamma_{j}\eta_{j}\cos2\gamma_{j}-2\gamma_{j}\eta_{j}\cosh2\beta_{j}-\\ \left.\frac{8\gamma_{j}\left(\gamma_{j}^{2}-\beta_{j}^{2}\eta_{j}^{2}\right)\cosh\beta_{j}\sin\gamma_{j}}{\beta_{j}^{2}+\gamma_{j}^{2}}-\gamma_{j}\left(\eta_{j}^{2}-1\right)\sin2\gamma_{j}+8\gamma_{j}\cos\gamma_{j}\left(\eta_{j}\cosh\beta_{j}-\frac{\beta_{j}\gamma_{j}\left(1+\eta_{j}^{2}\right)\sinh\beta_{j}}{\beta_{j}^{2}+\gamma_{j}^{2}}\right)+\frac{\left(\gamma_{j}^{2}+\beta_{j}^{2}\eta_{j}^{2}\right)\sinh2\beta_{j}}{\beta_{j}}\right). \end{array}$$

Dividing Equation (27) by $M_{j}$ and adding viscous damping with modal damping ratio of $\xi_{j}$ (A damping term is added to the equations at this point to keep the derivations simple), we have

(35) $${\ddot{q}}_{j}\left(t\right)+2\xi_{j}\omega_{j}{\dot{q}}_{j}\left(t\right)+\omega_{j}^{2}q_{j}\left(t\right)=\frac{P_{j}}{M_{j}}\left(t\right),\;\mathrm{for}\;j=1\ldots \infty$$

where $\omega_{j}^{2}=\frac{K_{j}}{M_{j}}$ is the j-th undamped natural frequency of the beam, as previously introduced in Equation (20). As mode shapes are arbitrary scaled (see Equation (22)), we use mass-normalized mode shapes to remove $M_{j}$ from equation above. To do so, we define $\phi_{j}\left(x\right)=\frac{\varphi_{j}\left(x\right)}{\sqrt{M_{j}}}$, and rewrite Equation (35) as

(36) $${\ddot{q}}_{j}\left(t\right)+2\xi_{j}\omega_{j}{\dot{q}}_{j}\left(t\right)+\omega_{j}^{2}q_{j}\left(t\right)=P_{j}\left(t\right),\;\mathrm{for}\;j=1\ldots \infty ,$$

in which $P_{j}\left(t\right)$ is calculated using Equation (30) after replacing $\varphi_{j}\left(x\right)$ with $\phi_{j}\left(x\right)$. The solution to Equation (36) can be obtained using the Duhamel integral [61] as $q_{j}\left(t\right)=\int_{0}^{t}h_{j}\left(t-\tau \right)P_{j}\left(t\right)d\tau$ where $h_{j}\left(t\right)$ is the continuous-time Impulse Response Function (IRF), defined as

(37) $$h_{j}\left(t\right)=\frac{1}{\omega_{jd}}e^{-\xi_{j}\omega_{j}t}\sin\omega_{jd}t,\;\mathrm{for}\;j=1\ldots \infty ,$$

where $\omega_{jd}=\omega_{j}\sqrt{1-\xi_{j}^{2}}$. The final solution, i.e., the lateral displacement of the beam at level x and time t, can be calculated as

(38) $$u\left(x,t\right)≅\sum_{j=1}^{m}\phi_{j}\left(x\right)q_{j}\left(t\right),$$

where the upper bound is restricted to include only the first m significant modes.

Using Equation (38), correlation between beam responses at two points $x_{1}$ and $x_{2}$ can be calculated as

(39) $$k_{u,u}\left(x_{1},x_{2},t\right)≅\sum_{k=1}^{m}\sum_{j=1}^{m}\phi_{k}\left(x_{1}\right)\phi_{j}\left(x_{2}\right)\;k_{q_{k},q_{j}}\left(t,t\right),$$

where $k_{q_{k},q_{j}}\left(t,t\right)$ is the correlation between modal coordinates $q_{k}\left(t\right)$ and $q_{j}\left(t\right)$, which can be calculated as

(40) $$k_{q_{k},q_{j}}\left(t,t\right)=\int_{0}^{t}\int_{0}^{t}k_{P_{k},P_{j}}\left(\tau_{1},\tau_{2}\right)h_{k}\left(t-\tau_{1}\right)h_{j}\left(t-\tau_{2}\right)d\tau_{1}d\tau_{2},$$

in which $k_{P_{k},P_{j}}\left(\tau_{1},\tau_{2}\right)$ is the correlation between modal loads $P_{k}\left(t\right)$ and $P_{j}\left(t\right)$. Similar to Papadimitriou et al. [43], we assume that the lateral load can be expressed in a separable form p(x,t)=s(x)r(t). Here, we further assume that the load is spatially deterministic and constant along the length of the beam, i.e., $s\left(x\right)=s_{0}$, so the only source of randomness arises from its time variation, r(t). Assuming this temporal randomness follows a zero-mean, stationary (constant statistical properties over time) Gaussian white noise process with a variance of $\sigma_{r}^{2}\delta \left(t_{1}-t_{2}\right)$, $k_{P_{k},P_{j}}\left(t_{1},t_{2}\right)$ can be calculated as

(41) $$k_{P_{k},P_{j}}\left(t_{1},t_{2}\right)={\hat{s}}_{k,j}\sigma_{r}^{2}\delta \left(t_{1}-t_{2}\right)\;,$$

where ${\hat{s}}_{k,j}=s_{0}^{2}\left(\int_{0}^{1}\phi_{k}\left(x\right)dx\right)\left(\int_{0}^{1}\phi_{j}\left(x\right)dx\right)$ in which

(42) $$\sqrt{M_{j}}\int_{0}^{1}\phi_{j}\left(x\right)dx=\frac{1-\cos\gamma_{j}}{\gamma_{j}}-\frac{\gamma_{j}}{\beta_{j}^{2}}\left(\cos\beta_{j}-1\right)+\eta_{j}\left[\frac{\sinh\beta_{j}}{\beta_{j}}-\frac{\sin\gamma_{j}}{\gamma_{j}}\right].$$

Therefore, when the modal responses reach stationary conditions (for large t), $k_{q_{k},q_{j}}$ can be calculated as

(43) $$k_{q_{k},q_{j}}={\hat{s}}_{k,j}\frac{2\nu_{k,j}}{{\left(\omega_{kd}^{2}-\omega_{jd}^{2}\right)}^{2}+\nu_{k,j}^{4}+2\nu_{k,j}^{2}\left(\omega_{kd}^{2}+\omega_{jd}^{2}\right)}\sigma_{r}^{2},$$

where $\nu_{k,j}=\xi_{k}\omega_{k}+\xi_{j}\omega_{j}$. Thus, the correlation between beam responses at two points $x_{1}$ and $x_{2}$ under stationary conditions becomes

(44) $$k_{u,u}\left(x_{1},x_{2}\right)≅\sum_{k=1}^{m}\sum_{j=1}^{m}\phi_{k}\left(x_{1}\right)\phi_{j}\left(x_{2}\right)\;k_{q_{k},q_{j}}.$$

This covariance kernel can be used in Equations (8) and (9) to predict the posterior mean and variance of the response at an unmeasured point $\hat{x}$ using information from measurement points $\bar{x}$, paving the way for optimal sensor placement, as will be further described in the next section.

### 2.3. The Objective Function

As mentioned earlier, the purpose of this study is to develop a method for determining the optimal sensor locations that result in a more reliable reconstruction of the response of non-instrumented floors. Gaussian Process Regression is a powerful non-parametric approach that can be used to achieve this objective. To maintain consistency, the optimal sensor locations are determined by minimizing the uncertainty in the GPR prediction, following the approach used by other researchers (e.g., [53]). Assuming there are $n_{d}$ sensors to be distributed along the height of the building, with normalized height $x\in \left[0,1\right]$, the optimal location of sensors are given by $x^{*}={\left[\begin{array}{cccc} {{\bar{x}}^{*}}_{1} & {{\bar{x}}^{*}}_{2} & \ldots & {{\bar{x}}^{*}}_{n_{d}} \end{array}\right]}^{T}$ and is obtained by solving the following minimization problem:

(45) $$x^{*}=\underset{\bar{x}\in S}{\min}\int \left(\left.\sigma_{f\left(\hat{x}\right)}^{2}\right|\bar{x}\right)d\hat{x},$$

where $\bar{x}$ is the vector of instrumented locations, S is the collection of all feasible/allowable points for instrumentation, and $\sigma_{f\left(\hat{x}\right)}^{2}$ is the posterior variance of the response at non-instrumented levels calculated using Equation (9) and the covariance kernel constructed in the previous section. For practical implementation, the objective function in Equation (45) can be expressed as a summation as follows

(46) $$OF=\frac{1}{n_{0}}\sum_{k=1}^{n_{0}}\left.\sigma_{f\left({\hat{x}}_{k}\right)}^{2}\right|\bar{x},$$

where ${\hat{x}}_{k}$ for $k=1\ldots n_{0}$ represents the normalized height of the building’s floors with $n_{0}$ levels. The optimization is carried out in Matlab [62] using the derivative-free search method fminsearch [63]. To enforce bound constraints (0≤x≤1), a transformation (see, e.g., [64]) is applied to convert the bounded problem into an unconstrained one that fminsearch can solve. Although the optimization should ideally be carried out in the discrete domain, we keep it continuous and convert the optimal solution $x^{*}$ to real-life by selecting the nearest floors.

As shown in Equation (9), response data is not required to compute $\sigma_{f\left({\hat{x}}_{k}\right)}^{2}$; therefore, the optimal sensor locations can be determined prior to sensor deployment, which is a key advantage of the proposed objective function. The only required information pertains to the characteristics of the beam model, which are necessary to compute the covariance kernel using Equation (44). Specifically, the parameters that contribute to the objective function include $s_{0}$, $\sigma_{r}^{2}$, α, ρ, $\omega_{j}$ and $\xi_{j}\;(for\;j=1\ldots m)$. From Equation (20), it is evident that once the first natural frequency $\omega_{1}$ is known, all other natural frequencies can be derived as

(47) $$\omega_{k}^{2}=\frac{\gamma_{k}^{2}\left(\alpha^{2}+\gamma_{k}^{2}\right)}{\gamma_{1}^{2}\left(\alpha^{2}+\gamma_{1}^{2}\right)}\;\omega_{1}^{2},$$

in which α is the only required parameter. Once α is known, the mode shapes can also be determined up to a free scaling factor. To obtain mass-normalized mode shapes, the mass density ρ is needed. However, since ρ introduces a constant scaling factor across all modes, it uniformly scales $k_{u,u}\left(x_{1},x_{2}\right)$ at all locations. The same applies to the load parameters $s_{0}$ and $\sigma_{r}^{2}$ (see Equation (43)). Therefore, it can be assumed that ${\hat{s}}_{k,j}\propto \left(\int_{0}^{1}\phi_{k}\left(x\right)dx\right)\left(\int_{0}^{1}\phi_{j}\left(x\right)dx\right)$. As a result, the only remaining controlling parameters are $\omega_{1}$, α and $\xi_{j}\;(for\;j=1\ldots m)$. It is reasonable to assume that modal damping ratios are of similar order across all modes for the purpose of optimal sensor placement, particularly when no structural response data is available. Based on this assumption, $k_{q_{k},q_{j}}$ can be further simplified as

(48) $$k_{q_{k},q_{j}}\propto {\hat{s}}_{k,j}\frac{1}{\omega_{1}^{3}}\frac{2\xi \left(b_{k}+b_{j}\right)}{\left(1-\xi^{2}\right){\left(b_{k}^{2}-b_{j}^{2}\right)}^{2}+\xi^{4}{\left(b_{k}+b_{j}\right)}^{4}+2\xi^{2}\left(1-\xi^{2}\right){\left(b_{k}+b_{j}\right)}^{2}\left(b_{k}^{2}+b_{j}^{2}\right)},$$

where $b_{k}=\gamma_{k}^{2}\left(\alpha^{2}+\gamma_{k}^{2}\right)/\gamma_{1}^{2}\left(\alpha^{2}+\gamma_{1}^{2}\right)$. This expression indicates that $\omega_{1}$ affects all auto- and cross-modal correlations in a similar manner and can therefore be omitted when computing from the objective function of Equation (46). Furthermore, assuming small damping (approximately 5%), Equation (48) can be simplified as

(49) $$k_{q_{k},q_{j}}\propto {\hat{s}}_{k,j}\frac{2\xi }{\left(b_{k}+b_{j}\right)\left[\left(1+2\xi^{2}\right)\left(b_{k}^{2}+b_{j}^{2}\right)-2b_{k}b_{j}\right]}.$$

When k=j, $k_{q_{k},q_{k}}\propto {\hat{s}}_{k,j}\frac{1}{4b_{k}^{3}\xi }$, indicating that changes in the damping ratio affect the modal coordinate autocorrelations in a uniform manner. Therefore, we can write: $k_{q_{k},q_{k}}\propto {\hat{s}}_{k,k}\frac{1}{4b_{k}^{3}}$. For k≠j, the $2\xi^{2}$ term can be neglected in comparison to 1 and express: $k_{q_{k},q_{j}}\propto {\hat{s}}_{k,j}\frac{2\xi }{\left(b_{k}+b_{j}\right){\left(b_{k}-b_{j}\right)}^{2}}$. Here, damping appears again as a scaling factor affecting all cross-correlations similarly, though with a different order than in the autocorrelation case. However, since modal cross-correlations are generally much smaller than autocorrelations, the damping ratio can also be excluded from the list of controlling parameters. Thus, the only remaining parameter is α, which characterizes the relative contribution of flexural and shear behavior and can be estimated based on the building’s lateral force-resisting system. For example [26]:

- In shear wall and braced frame buildings, α typically ranges between 0 and 1.5.
- In dual structural systems—such as a combination of moment-resisting frames with shear walls or braced frames—α usually falls between 1.5 and 5.
- In moment-resisting frame buildings, α typically ranges from 5 and 20.

**Figure 4:**
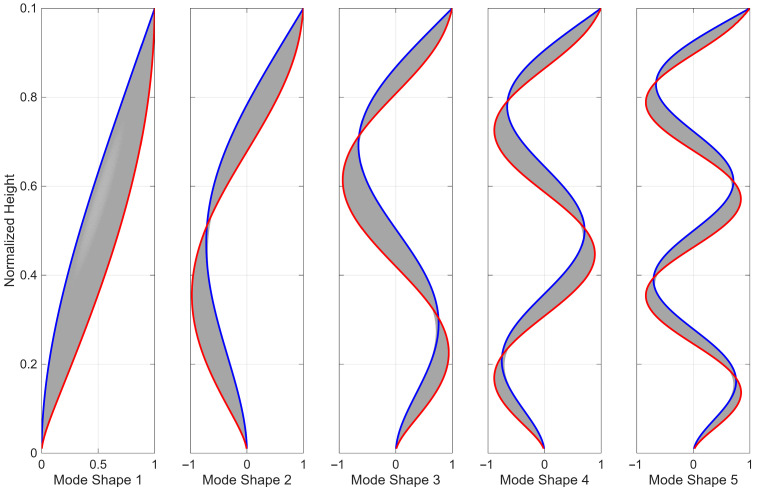
The first five mode shapes of the coupled beam model with α varying from 0 to 30. The blue curve represents pure flexural behavior, while the red curve represents pure shear behavior.

Figure 4 shows the first five mode shapes of the coupled beam model with α ranging from 0 to 30. The blue curve represents pure flexural behavior, while the red curve represents pure shear behavior. As shown, the nodes in each mode move toward the base of the beam as the behavior transitions from flexural to shear dominance. Additionally, the differences between mode shapes diminish for higher modes.

Finally, the overall workflow of the proposed optimal sensor placement methodology is summarized in Table 1 as pseudocode. 

## 3. Case Studies

### 3.1. Single Sensor

**Figure 5:**
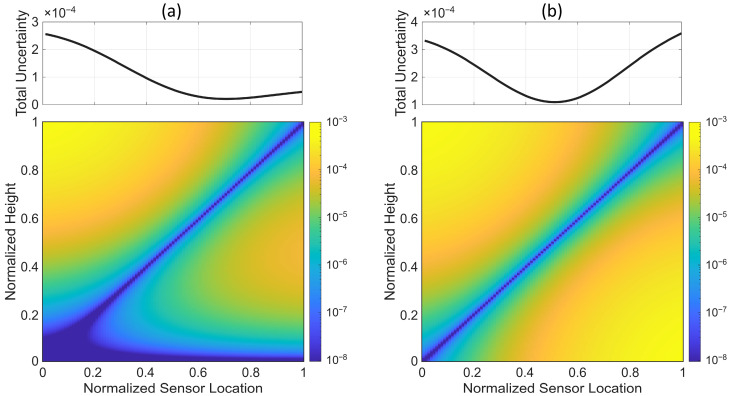
Response variance and total uncertainty versus the location of a single sensor for a building with α=10. (**a**) Response variances are normalized by the roof’s prior variance, and (**b**) response variances are normalized by their respective prior variances.

First, we present the optimal sensor placement when only one sensor is deployed. While more than one sensor is usually used in real applications, this single-sensor scenario helps to understand and verify the details of the methodology. Figure 5 shows the results for a case with α=10. In Figure 5a, the response variance at each point along the height of the beam ($\sigma_{f\left(\hat{x}\right)}^{2}$ in Equation (9)) is shown versus the location of the sensor. In this heatmap plot, all responses are normalized to the prior variance of the roof, $k\left(1,1\right)$, to eliminate the effects of load intensity on the absolute variance values for comparison purposes. We will refer to this scaling scheme as uniform scaling in this paper. As can be seen, the variance is close to zero along the diagonal because the sensor is placed there, and it increases as the response point moves farther from the measurement points—especially when the response point is close to the roof while the sensor is placed near the base. The objective function defined in Equation (46) is computed by averaging along the vertical direction of the heatmap plot and is shown in the top subplot. As seen, the minimum total uncertainty is obtained when the single sensor is placed at a normalized height of 0.71.

**Figure 6:**
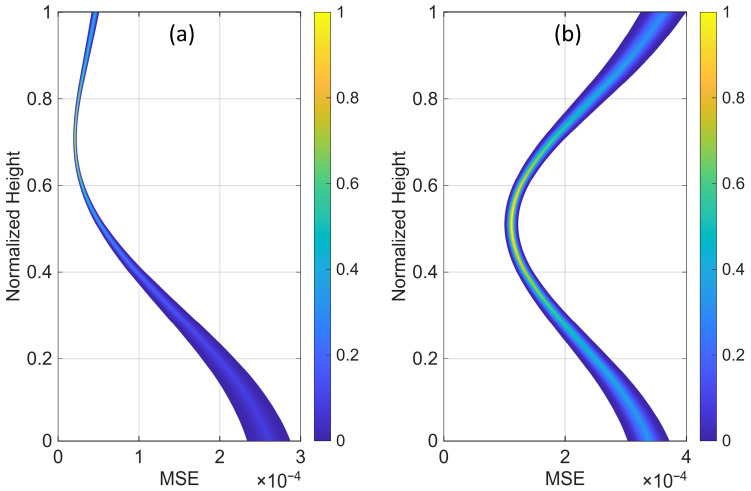
Probability Distribution Function (PDF) of the Mean Squared Error (MSE) of the beam’s total response versus the location of a single sensor in a building with α=10. (**a**) Response errors are normalized by the MSE at the roof (uniform scaling), and (**b**) response errors are normalized by the MSE of their respective floors (nonuniform scaling).

Figure 6b presents similar plots, but with each response variance scaled by its prior variance (i.e., $\sigma_{f\left(\hat{x}\right)}^{2}/k\left(\hat{x},\hat{x}\right)$). In this paper, we will refer to this scaling scheme as nonuniform scaling. With this normalization, the response uncertainty changes uniformly around the sensor location. Consequently, the total uncertainty reaches its minimum when the sensor is positioned at the mid-height of the building, as shown in the top subplot of Figure 6b.

To further evaluate the performance of this optimal sensor placement strategy, we subjected the beam model with α=10 and a 5% modal damping ratio to 500 randomly generated white noise realizations, ensuring a sufficient duration to achieve a stationary response. Assuming sensor single-sensor measurements, we then predicted the mean responses along the height of the beam for each case using Equation (8). Finally, we calculated the Mean Squared Error (MSE) of the predictions for two scaling scenarios as follows

(50) $$MSE(\bar{x})=\frac{1}{n_{0}}\sum_{k=1}^{n_{0}}\frac{\sum_{n=1}^{n=N}{\left[u_{m}\left({\hat{x}}_{k},n\right)-u({\hat{x}}_{k},n)\right]}^{2}}{\sum_{n=1}^{n=N}{u(1,n)}^{2}},$$

(51) $$MSE(\bar{x})=\frac{1}{n_{0}}\sum_{k=1}^{n_{0}}\frac{\sum_{n=1}^{n=N}{\left[u_{m}\left({\hat{x}}_{k},n\right)-u({\hat{x}}_{k},n)\right]}^{2}}{\sum_{n=1}^{n=N}{u({\hat{x}}_{k},n)}^{2}},$$

where $u({\hat{x}}_{k},n)$ and $u_{m}\left({\hat{x}}_{k},n\right)$ are, respectively, the true and mean predicted response at the normalized level ${\hat{x}}_{k}$ and time instant n. N is the length of the signals, and $n_{0}$ is the total number of discrete floors, as introduced earlier.

**Figure 7:**
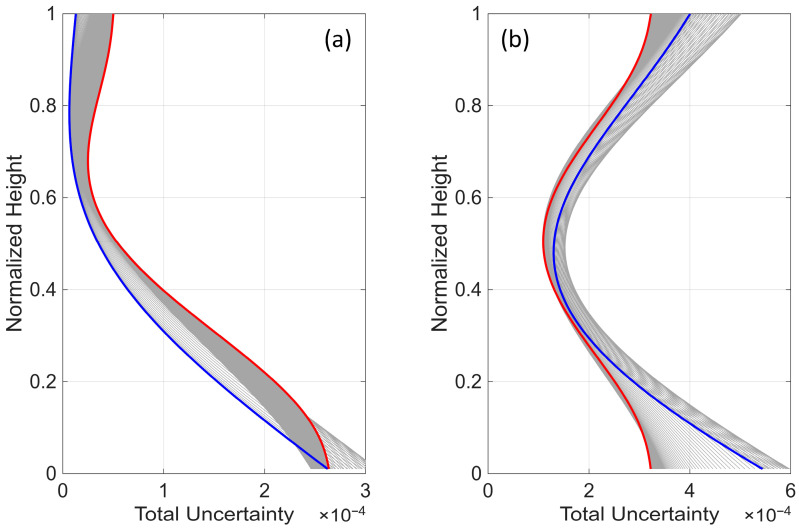
Variation of the total uncertainty with sensor location for beams with α ranging from 0 to 30. The blue curve represents pure flexural behavior, while the red curve represents pure shear behavior: (**a**) uniform scaling and (**b**) nonuniform scaling.

**Figure 8:**
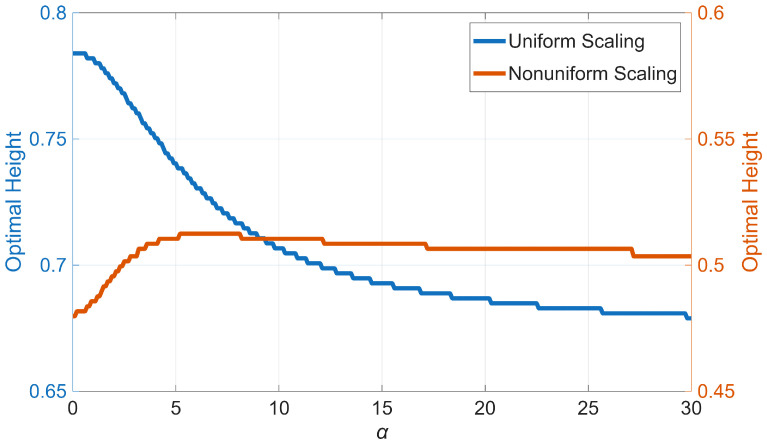
Variation of the optimal sensor location with α.

Figure 6 shows the Probability Distribution Function (PDF) of the MSE versus the location of a single sensor along the height of the beam for the two scaling scenarios. As expected, the mean MSE is minimized when the sensor is placed at a height of 0.71 when the roof variance is used as the scaling factor. In contrast, the mid-height of the beam is the optimal sensor location when each floor’s MSE is scaled by its response variance. Additionally, both the mean MSE and its uncertainty are minimized when the sensor is at the optimal location, as seen in both plots of Figure 6. Finally, we repeat the optimal sensor placement analysis for the entire range of the parameter α (i.e., 0 to 30). The total uncertainty for all of these cases and both scaling scenarios is shown in Figure 7. Similar to the mode shape plots (Figure 4), cases with pure flexural and pure shear behaviors are highlighted in blue and red, respectively. As observed, when the variances are not scaled (or scaled using a constant, such as the roof’s variance), the optimal sensor location shifts from higher to lower levels as the beam behavior transitions from flexural to shear-dominated. However, under the second scaling scheme, the optimal location remains nearly unchanged and even shifts slightly upward. While changes in the optimal location versus α are generally negligible in this case, the total uncertainty obtained from the two most extreme cases (i.e., pure shear and pure flexural) does not perfectly represent the lower and upper envelopes of all cases. Additionally, the order of the blue and red curves is inverted in Figure 7b compared to Figure 7a because, when scaling each floor’s uncertainty by its prior variance, all floors contribute equally to the total uncertainty. As a result, the total uncertainty is smaller for the pure shear beam, where the lateral displacement is nearly uniform along the height of the structure, compared to the pure bending beam, where the response gradually increases from bottom to top. Figure 8 presents the optimal sensor location versus α for both scaling scenarios. As mentioned earlier and shown in this figure, when nonuniform scaling is employed, the optimal location remains nearly unchanged. However, with uniform scaling, the optimal location shifts to lower elevations as the shear contribution increases.

### 3.2. Multiple Sensors

**Figure 9:**
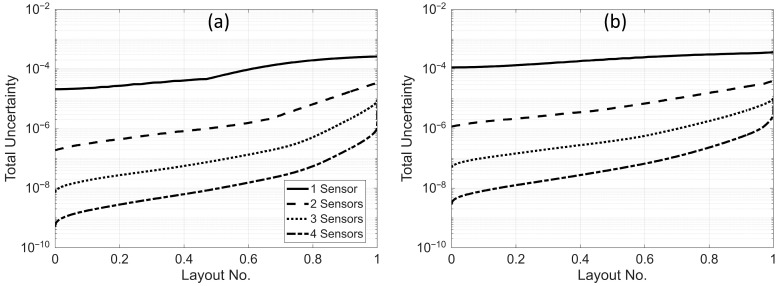
Variation of total uncertainty of a beam with α=10 for different numbers of sensors under (**a**) uniform scaling and (**b**) nonuniform scaling scenarios.

When the number of sensors is greater than one, it may not be possible to graphically present some of the results shown in the previous section. Figure 9 illustrates the total uncertainty (objective function) of a beam with α=10 versus a normalized index representing the sensor layout. For each sensor count, we calculated the objective function for all possible sensor arrangements within the available $n_{0}$ locations, then sorted the resulting objective values from minimum to maximum. Because the number of possible combinations varies with the number of sensors, we normalized the sorted layout indices (ranging from 1 to the total number of combinations) by the maximum number of combinations to enable plotting all cases on the same figure.

The single-sensor scenario in Figure 9 corresponds to the top plots of Figure 5, now simply sorted from minimum to maximum. As seen in Figure 9, adding a second sensor significantly reduces the objective function, regardless of the sensor locations. This reduction can reach up to two orders of magnitude if the sensors are optimally placed. The figure demonstrates that, under both scaling schemes, using two sensors instead of one results in lower total uncertainty—even if the sensors are randomly located in the building. This trend of decreasing total uncertainty continues with the addition of more sensors, as shown in Figure 9. The number of sensors is limited to four in this figure because only five modes are considered in the analysis; thus, using five sensors would eliminate the uncertainty entirely. This figure can also be used to determine both the number and locations of sensors required to achieve a specified level of total uncertainty.

**Figure 10:**
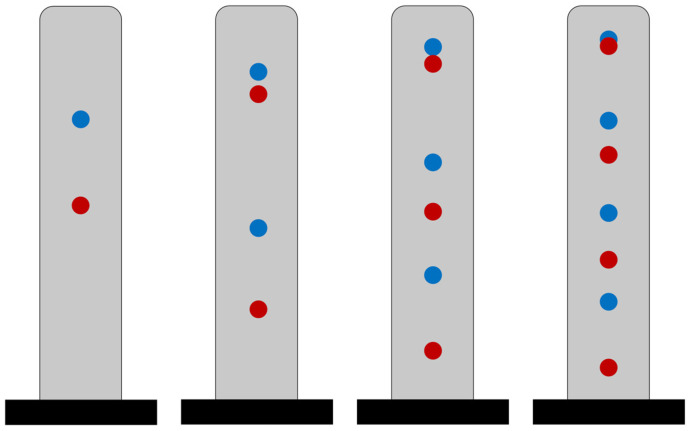
Optimal sensor locations for a beam with α = 10, shown for varying numbers of sensors under uniform (blue) and nonuniform (red) scaling scenarios.

Figure 10 shows the optimal sensor locations corresponding to the minimum objective function. In this figure, blue circles indicate the optimal locations under the uniform scaling scenario, while red circles represent the optimal locations under the nonuniform scaling scenario. As seen, and similar to the single-sensor case, scaling the response variances by their corresponding prior variances results in a downward shift in the optimal sensor locations.

**Figure 11:**
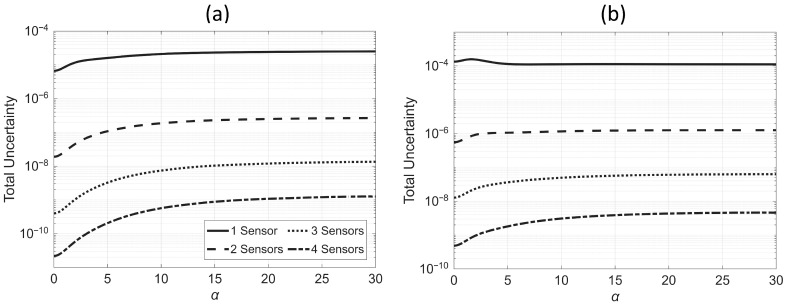
Variation of the minimum total uncertainty with α for different numbers of sensors: (**a**) uniform scaling and (**b**) nonuniform scaling.

Similar to the single-sensor scenario, we now solve the problem for a range of α values, varying from zero (pure flexural beam) to 30 (pure shear beam). Figure 11 shows the minimum total uncertainty for different numbers of sensors under both uniform and nonuniform scaling scenarios. As seen in the figure, for both scaling cases, once α exceeds a certain threshold (~15 for uniform scaling and ~10 for nonuniform scaling), the minimum total uncertainty becomes almost insensitive to further changes in α. In other words, the minimum total uncertainty is more sensitive to the balance between shear and flexural contributions when the beam’s behavior is dominated by flexural effects—typical of buildings with concrete shear walls.

**Figure 12:**
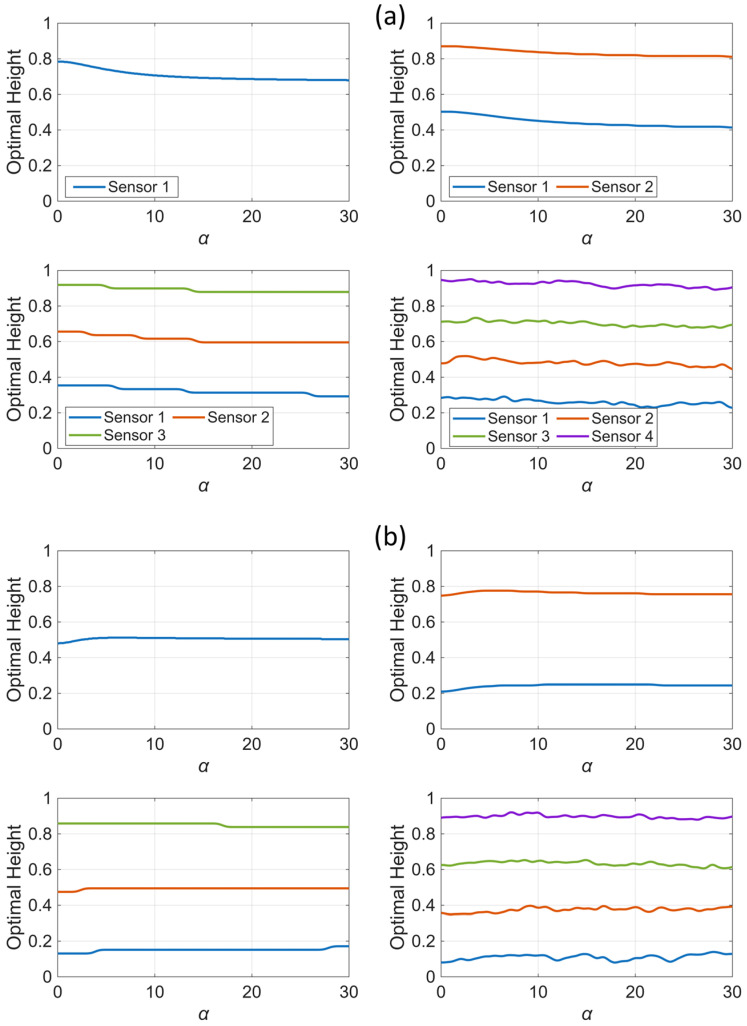
Variation of the optimal sensor location with α for different numbers of sensors under (**a**) uniform and (**b**) nonuniform scaling. The small fluctuations and step-like patterns observed in the figure are due to the limited discretization resolution used in the analysis and the nonconvex nature of the optimization problem.

Figure 12 shows the optimal sensor heights for various numbers of sensors under both uniform and nonuniform scaling scenarios. As observed in the single-sensor case, when the building behavior shifts toward shear behavior, the optimal sensor locations move toward the lower levels. Additionally, and consistent with the observations in Figure 10 for a fixed value of α, nonuniform scaling further shifts the optimal sensor locations downward and arranges them in a symmetric pattern with respect to the building’s mid-height.

To make the results of this study applicable to engineering practice, we recommend optimal sensor locations for three common structural systems, as shown in Table 2, based on the mean α values suggested by [26], under both scaling scenarios. In the absence of detailed information about a specific building, if the structure can be reasonably approximated by a beam model, this table can guide sensor placement along the building height to enable estimation of non-instrumented floor response using Gaussian Process Regression with minimal total uncertainty. To use this table, the normalized sensor heights—based on the building’s lateral system and the total number of sensors—should be multiplied by the total height of the building. The floor closest to the calculated physical height is then identified.

## 4. Validation

**Figure 13:**
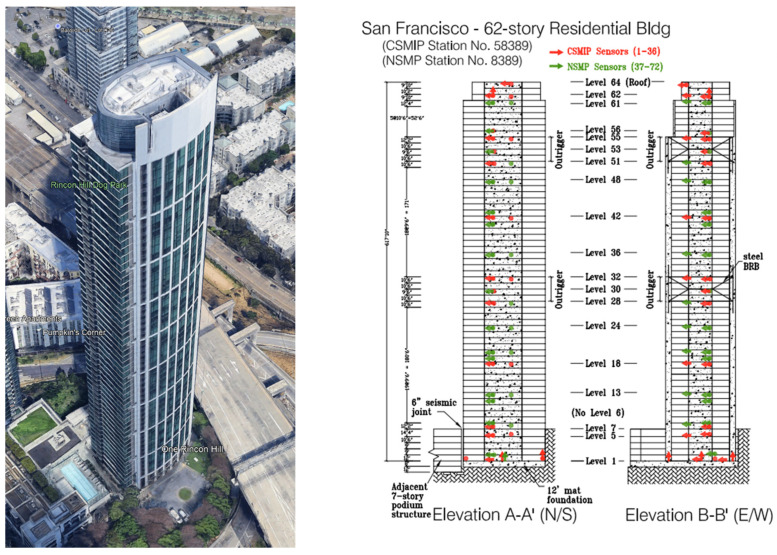
One Rincon Hill building (**left**) and its instrumentation layout (**right**).

In this section, data from an instrumented building is used to validate the optimal sensor placement method described and tested in the previous sections. A complete validation ideally requires a tall building instrumented on every floor, with data that align with the load assumptions made during the method’s derivation. Unfortunately, to the best of the authors’ knowledge, no such building exists in the CSMIP database. One of the best available cases for validation is the One Rincon Hill (ORH) building in San Francisco (see Figure 13). This 62-story building features a concrete core shear wall supplemented by tall outrigger columns. The core is connected to the outrigger columns using steel buckling-restrained braces. Additionally, the building is equipped with rooftop water tanks that function as liquid mass dampers. The floors consist of post-tensioned concrete flat slabs, and the building is supported by a 12-ft-thick mat foundation resting on bedrock. The ORH building was instrumented in 2012 through a joint effort by the California Geological Survey (CGS) and the United States Geological Survey (USGS), using 72 accelerometers across 26 levels. Figure 13 illustrates the existing instrumentation, with half of the sensors managed by CGS and the other half by USGS.

**Figure 14:**
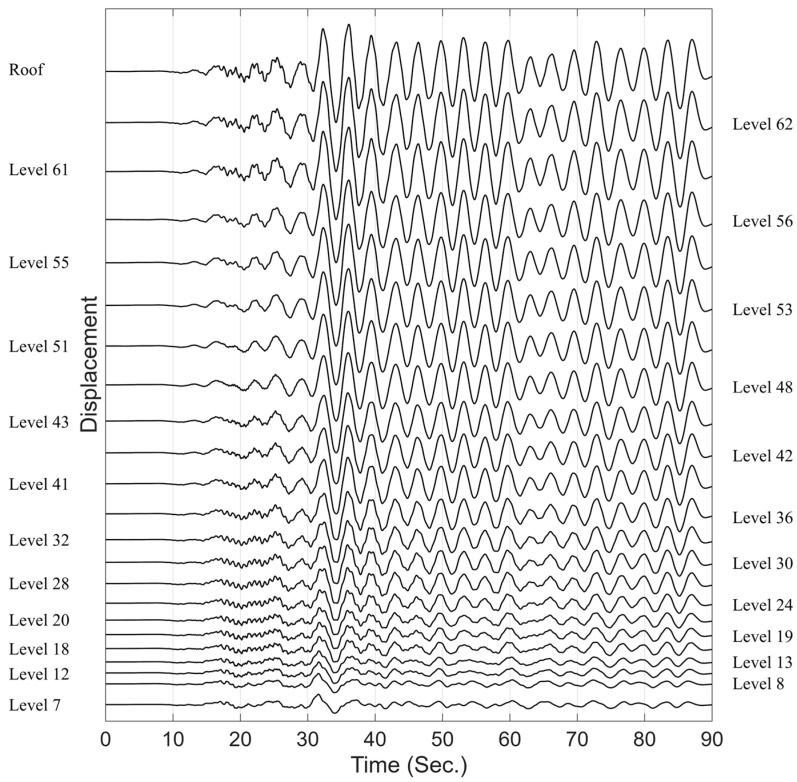
Recorded displacement response during the 2014 M6 South Napa earthquake, from Level 7 up to the roof.

While some publicly available stationary ambient data exist, we use one of the weak earthquake records available through CESMD (Center for Engineering Strong Motion Data) (strongmotioncenter.org, last updated: 15 May 2025) for validation to ensure that the applied lateral inertia forces are spatially deterministic and uniform. The 2014 M6 South Napa earthquake, which occurred approximately 50 km from the building, produced a Peak Ground Acceleration (PGA) of 0.005 g. This level of ground motion satisfies the assumption of stationarity and temporal uncorrelation used in the formulation. Figure 14 shows the recorded displacement signals (NS direction) used to identify optimal sensor locations for this building under different scaling and sensor quantity scenarios. Since the building’s floor plan changes significantly at Level 7, we consider that level as the base of the structure for calculating floor heights and selecting data channels. This results in a total of 23 instrumented floors used in the analysis.

The 90-s displacement data used in this example were obtained by numerical integration of acceleration records measured using EpiSensor ES-U2 force-balance accelerometers (Kinemetrics, Pasadena, USA) [65] at a sampling rate of 100 Hz. More details on the sensor specifications and the minimum requirements for the types of sensors used in building instrumentation can be found in references such as [66].

**Figure 15:**
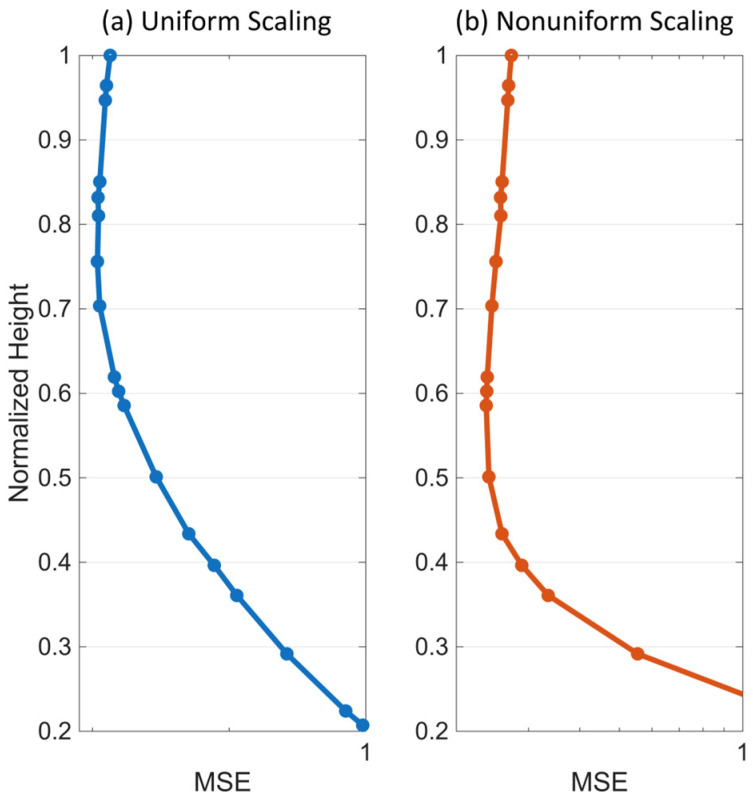
Variation of the mean-square error with the location of a single sensor along the height of the building for (**a**) uniform and (**b**) nonuniform scaling scenarios.

To verify the findings reported in Table 2, we predict the mean response of the non-instrumented floors under each scaling and sensor quantity scenario using Equation (8), and then compute the errors using Equations (50) and (51). The minimum error is expected when the sensor is placed at the optimal location. Figure 15 shows the variation of the mean-square error for the single-sensor scenario. This figure closely resembles Figure 6, although it is based on real data from a building that does not perfectly behave as a shear-flexural beam (an α value of 1.5 is used in the beam model calculations). Also, the loading is not ideal stationary white noise. As seen in the figure, the optimal sensor locations are approximately 0.83 and 0.58 times the building height for the uniform and nonuniform scaling scenarios, respectively, consistent with the results suggested in Table 2.

**Figure 16:**
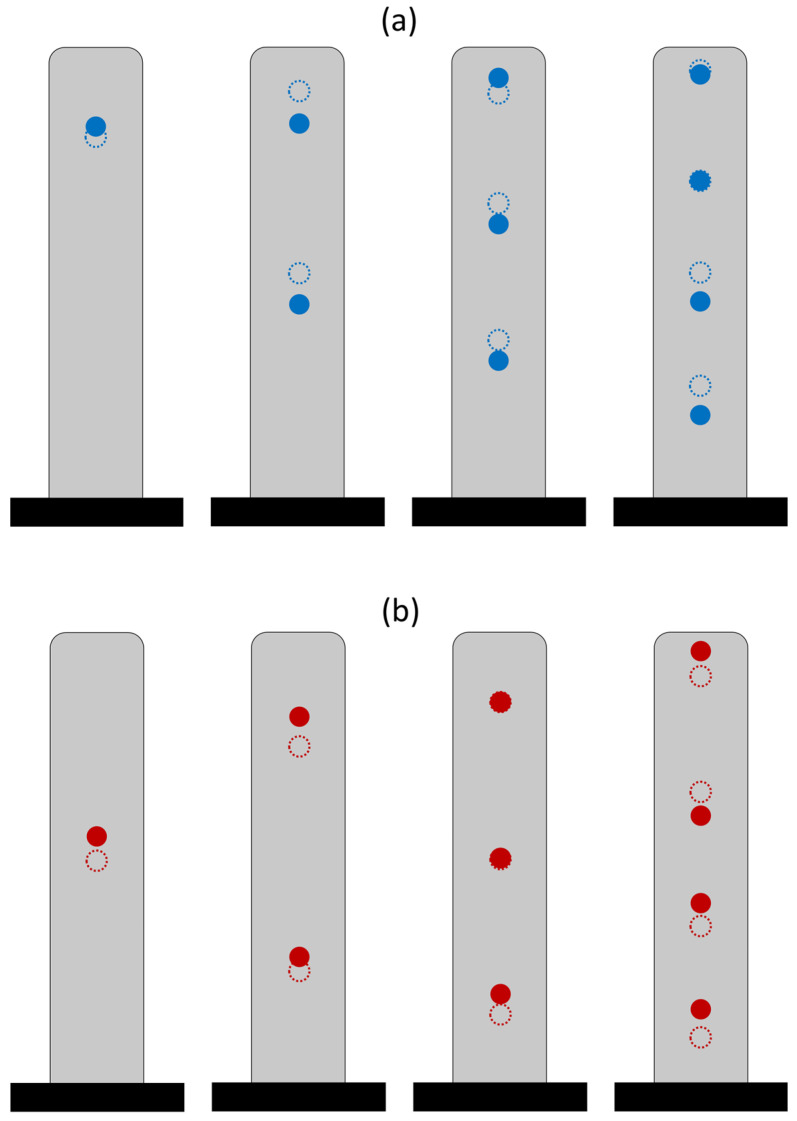
Identified optimal sensor locations (filled circles) for the ORH building compared to the locations suggested in Table 1 (unfilled circles), shown for varying numbers of sensors under (**a**) uniform (blue) and (**b**) nonuniform (red) scaling scenarios.

Figure 16 displays the identified optimal sensor locations for various numbers of sensors under the two scaling scenarios. As shown in the figure, the identified sensor locations closely match those suggested in Table 2 across different sensor quantities.

While the numerical examples used for verification satisfy all the assumptions considered in deriving the optimal sensor placement methodology (uniform mass and stiffness, stationary signals, one-dimensional response, absence of soil–structure interaction effects, etc.), the validation example in this section demonstrates that the proposed methodology remains effective under real-life conditions where these assumptions may be partially violated. Furthermore, although the method presented in this study assumes a one-dimensional response, the proposed methodology remains applicable to buildings with rigid diaphragms and symmetric floor mass and stiffness distributions, where the three degrees of freedom at each floor are decoupled, and optimal sensor placement can be performed independently for each direction. However, if these conditions are not satisfied, the use of a one-dimensional beam model is no longer accurate. The treatment of such cases is beyond the scope of this paper.

## 5. Conclusions

We developed an optimal sensor placement methodology based on an objective function that minimizes the response reconstruction uncertainty at non-instrumented floors. The California Strong Motion Instrumentation Program (CSMIP) has been instrumenting civil engineering structures, including buildings and bridges, for over 50 years. One of the primary goals of this program is to provide high-quality structural response data to the engineering community. However, due to the high cost of sensor installation and maintenance, it is not feasible to densely instrument most structures. For example, many buildings are currently instrumented using engineering judgment, with only a few sensors distributed along their height. To make this process more quantitative and systematic, we developed a method for identifying optimal sensor locations along the height of building structures, enabling the interpolation of responses at non-instrumented floors with minimal uncertainty. The proposed method is limited to stationary excitations and buildings with uniform mass and stiffness distributions along their height. Under these assumptions, the kernel function of the Gaussian Process Regression (GPR) model can be analytically derived based on the shear-flexural beam model and random vibration theory. We verified and validated the proposed method using both simulated and real data. In addition, we proposed a table that can be used to support more quantitative and informed decision-making regarding sensor placement. This study shows that if the prediction uncertainty at all levels of the building is equally important (nonuniform scaling scenario), the optimal sensor locations along the height of the structure are typically lower than in the case of uniform scaling, where the higher levels exhibit larger responses. It is worth noting that although the assumptions adopted in this study may appear restrictive, they achieve a practical balance between accuracy and simplicity, making the approach suitable for large-scale applications such as CSMIP. Ongoing efforts aim to extend this framework to non-stationary excitations and a wider range of building types by training the GPR model on recorded seismic data instead of relying on random vibration theory.

## Figures and Tables

**Table 1:** The overall workflow of the proposed method.

Initialization
Set $\omega_{1}$, ρ$,\;s_{0}$ and $\sigma_{r}^{2}$ to arbitrary values
Set m and ξ based on engineering experience
Set α based on the lateral system of the building
Beam model
$\gamma_{j}$: numerically find the roots of the characteristic equation corresponding to Equation (17)
$\omega_{j}^{2}$: Equation (47)
$\omega_{jd}$ $:\;\omega_{j}\sqrt{1-\xi_{j}^{2}}$
$\varphi_{k}\left(x\right)$: Equation (22)
Parameters needed for the optimization
$\int_{0}^{1}\varphi_{j}^{2}\left(x\right)$: Equation (33)
$M_{j}$: Equation (28)
$\int_{0}^{1}\phi_{j}\left(x\right)$: Equation (42)
${\hat{s}}_{k,j}$ $:\;s_{0}^{2}\left(\int_{0}^{1}\phi_{k}\left(x\right)dx\right)\left(\int_{0}^{1}\phi_{j}\left(x\right)dx\right)$
$\nu_{k,j}$ $:\;\xi_{k}\omega_{k}+\xi_{j}\omega_{j}$
$k_{q_{k},q_{j}}$: Equation (43)
$\hat{x}$: normalized height of all floors
$\bar{x}$: normalized height of candidate instrumented floors
Optimization loop until $\bar{x}$ does not change
$\sigma_{f\left(\hat{x}\in x\right)}^{2}$: Equation (9)
$\;C\left(\bar{x},{\bar{x}}^{\prime }\right)$: Equation (5)
$\;k\left(\bar{x},\hat{x}\right)$: Equation (6)
$k\left(x_{1},x_{2}\right)$: Equation (44)
Scaling:
$\sigma_{f\left(\hat{x}\in x\right)}^{2}/k\left(1,1\right)$: Uniform scaling scenario
$\sigma_{f\left(\hat{x}\in x\right)}^{2}/k\left(\hat{x},\hat{x}\right)$: Nonuniform scaling scenario
Objective Function (OF): Equation (46)
Update $\bar{x}$

**Table 2:** Recommended optimal heights of sensors under uniform and nonuniform scaling scenarios for buildings with common structural systems. Numbers in parentheses are for the nonuniform scaling scenario.

	Shear Wall/Braced Frame				Dual System				Moment Frame			
	Number of Sensors				Number of Sensors				Number of Sensors			
Sensor Number	1	2	3	4	1	2	3	4	1	2	3	4
1	0.80 (0.50)	0.50 (0.25)	0.35 (0.15)	0.30 (0.1)	0.75 (0.50)	0.50 (0.25)	0.35 (0.15)	0.30 (0.1)	0.70 (0.5)	0.45 (0.25)	0.30 (0.15)	0.25 (0.1)
2		0.9 (0.75)	0.65 (0.50)	0.50 (0.35)		0.85 (0.75)	0.65 (0.50)	0.50 (0.35)		0.85 (0.75)	0.60 (0.50)	0.50 (0.35)
3			0.90 (0.85)	0.70 (0.65)			0.90 (0.85)	0.70 (0.65)			0.90 (0.85)	0.70 (0.65)
4				0.95 (0.90)				0.95 (0.90)				0.95 (0.90)

## Data Availability

All real earthquake data used in this study are publicly available at strongmotioncenter.org. All other simulated models, computer codes, and data are available from the first author upon reasonable request.

## Abbreviation

Nomenclature and definitions of symbols used in this study.

**Symbol**	**Description**
GP(.)	Gaussian Process
$E\left[.\right]$	Expected value
k	Kernel function
C	Covariance matrix
$\sigma_{0}^{2}$	Measurement noise variance
$N(\mu ,\sigma^{2})$	Normal distribution with μ and variance $\sigma^{2}$
$n_{d}$	Number of data points/instrumented floors
ρ	Mass per unit length
GA	Shear stiffness
EI	Flexural stiffness
L	Beam length/building height
$\varphi_{j}$	j-th mode shape
$q_{j}$	j-th model coordinate
α	Dimensionless parameter
u	Lateral displacement
$M_{j}$	j-th modal mass
$K_{j}$	j-th modal stiffness
$P_{j}\left(t\right)$	j-th modal load
$\omega_{j}$	j-th undamped natural frequency
$\xi_{j}$	j-th modal damping ratio
$\phi_{j}$	j-th mass-normalized mode shape
$\omega_{jd}$	j-th damped natural frequency
$k_{q_{k},q_{j}}$	Modal coordinates cross-reaction
$k_{P_{k},P_{j}}$	Modal loads’ cross-correlation
$k_{u,u}$	Correlation response
$\bar{x}$	Vector of instrumented locations
$n_{0}$	Total number of floors
$x^{*}$	Vector of optimal locations
s(x)	Load spatial distribution
r(t)	Load temporal distribution
$\sigma_{r}^{2}$	Load temporal variance
N	Number of time samples
